# Serial testing of health care workers for tuberculosis infection: A prospective cohort study

**DOI:** 10.1371/journal.pone.0235986

**Published:** 2020-07-17

**Authors:** Irma Casas, Maria Esteve, Rosa Guerola, Irene Latorre, Raquel Villar-Hernández, Guillermo Mena, Cristina Prat-Aymerich, Joan Matllo, Jose Dominguez

**Affiliations:** 1 Servei de Medicina Preventiva, Hospital Universitari Germans Trias i Pujol, Barcelona, Spain; 2 Universitat Autònoma de Barcelona, Barcelona, Spain; 3 Servei de Microbiología, Hospital Universitari Germans Trias i Pujol, Institut d’Investigació Germans Trias i Pujol, Barcelona, Spain; 4 CIBER Enfermedades Respiratorias, CIBERES, Instituto de Salud Carlos III, Madrid, Spain; 5 Julius Center for Health Sciences and Primary Care, University Medical Center Utrecht, Utrecht University, Utrecht, The Netherlands; 6 Unitat de Salut Laboral, Hospital Universitari Germans Trias i Pujol, Institut Català de la Salut, Barcelona, Spain; The University of Georgia, UNITED STATES

## Abstract

Health Care Workers (HCW) may have an occupational risk of latent tuberculosis infection (LTBI) and TB disease. The objective of this study was to evaluate the performance of the 2-step strategy: tuberculin skin test (TST) followed by confirmation with Interferon (IFN)-γ- release assays (IGRAs) in HCW. A secondary objective was to determine the factors related to conversions and reversions. HCW at risk of occupational exposure who attended the Occupational Department of the Hospital Germans Trias i Pujol were included during the study period (2013–2016). All professionals testing negative for LTBI were included in a cohort study. These workers were followed up with the administration of a TST and an IGRA quantification at least one year after inclusion in the study. Workers with positive TST, regardless of the results of the IGRA tests, were followed-up with an IGRA. 255 workers were enrolled in the study and 108 workers from the same cohort were followed up. During the follow-up period, seven workers presented TST test conversion. One of these conversions was also confirmed by an IGRA test. There were 2 conversions of cases only testing positive with the IGRA. There have been only 2 reversions of cases testing negative with the IGRA. In this study, not all TST conversions were confirmed when using the IGRA test, which highlights the importance of the 2-step strategy. We have detected a low number of conversions and reversions. Our conclusions should be confirmed in studies with a longer follow-up time.

## Introduction

Tuberculosis (TB) continues to be a major worldwide public health problem [1]. Health Care Workers (HCW) can have an occupational risk of latent tuberculosis infection (LTBI) and TB disease [2]. International and national guides recommend that HCW undergo LTBI screening periodically to assess if they have a LTBI and treat it [3–5]. To do this, it is important to have valid and reliable diagnostic tests. Interferon (IFN)-(gamma)-γ- release assays (IGRAs) have been available for some time and they provide some advantages over the tuberculin skin test (TST). IGRAs have been evaluated in different populations (immunosuppressed, children, HIV infected, contact studies, etc.) and have been included in some of the guidelines [6]. Since 2010 [7], the CDC of the United States includes tests determined by IGRAs as alternative screening to TST and prioritizes its use in individuals vaccinated with BCG. Based on the risk of both infection and disease progression, the updated 2017 recommendations [8] suggested the use of IGRA tests in HCW with a moderate or high risk of infection and low or intermediate risk of disease progression and considered the TST as an acceptable alternative.

In our hospital, a study was carried out in 2005 [9] of 147 HCW with the objective of analyzing the concordance among the two available IGRAs techniques (QFN-G-IT and T. SPOT-TB) and TST. The results suggested that the first two tests detected more recent infections than the TST. Neither of the two IGRA techniques were affected by BCG vaccination. In a systematic review [10] of the use of IGRAs in HCW, the authors concluded that the use of IGRAs for serial testing is complicated by lack of data on optimum cut-offs and the unclear interpretation and prognosis of conversions and reversions. They also claimed that further longitudinal research would be required to inform guidelines on serial testing using IGRAs in HCW. Conclusions from subsequent cohort studies in low incidence settings [11, 12] did not help to clarify the role of conversions and reversions of IGRAs. Therefore, it seems prudent to screen for tuberculosis infection in HCW with the TST while the result is negative. In the case of a positive TST result, a 2-step strategy, using an IGRA to confirm the positivity, could provide more information in order to make an informed decision on the recommended treatment for LTBI [6, 13].

The diagnosis of tuberculosis infection in HCW, and especially in recent converters, results in a long-term treatment. Because of this, it is very important to have evidence to rule out any recent tuberculosis infection, so as not to expose the HCW to unnecessary treatments with possible side effects. At the same time this evidence would enable clinicians to better indicate treatment for those individuals for whom the benefit may be greater [4]. The objective of this prospective cohort study was to evaluate the performance of the 2-step strategy in HCW at a tertiary hospital. A secondary objective was to determine the factors related to conversions and reversions.

## Methods

### Study setting

The Hospital Universitari Germans Trias i Pujol (HUGTiP) is a tertiary, high-technology hospital of the Barcelona Nord i Maresme Health Region of Catalonia (Spain). The hospital has 530 beds and a workforce of 3.000. The HUGTiP is a reference hospital for more than 800.000 people. Over 50 new cases of TB are detected every year in the hospital. The incidence of TB in Catalonia is currently 13 per 100,000 inhabitants [14]

### Study design

#### Phase 1: Cross-sectional study

Medical Doctors, nurses, nursing assistants, health technicians, caretakers, research staff and others at risk of occupational exposure to *M*. *tuberculosis* and who attended at the Occupational Department of the HUGTiP were included during the study period, as were those who were tested for TST in the context of their health examination (either initial or periodic).

Those professionals with a history of prior positive TSTs, a history of TB or treatment, or of tuberculosis infection were excluded. HCW with some type of immunosuppression (basic diseases or immunosuppressive treatment) and pregnant workers were also excluded.

From October 2013 to December 2016, 255 workers were enrolled in the study.

#### Phase 2: Cohort study

Professionals without a tuberculosis infection (negative result of TST and IGRA tests in the first phase) and at risk of occupational exposure to *M*. *tuberculosis* were included in this phase. These workers were followed up with TST administration and IGRA quantification at least one year after inclusion in the study.

**Fig 1:**
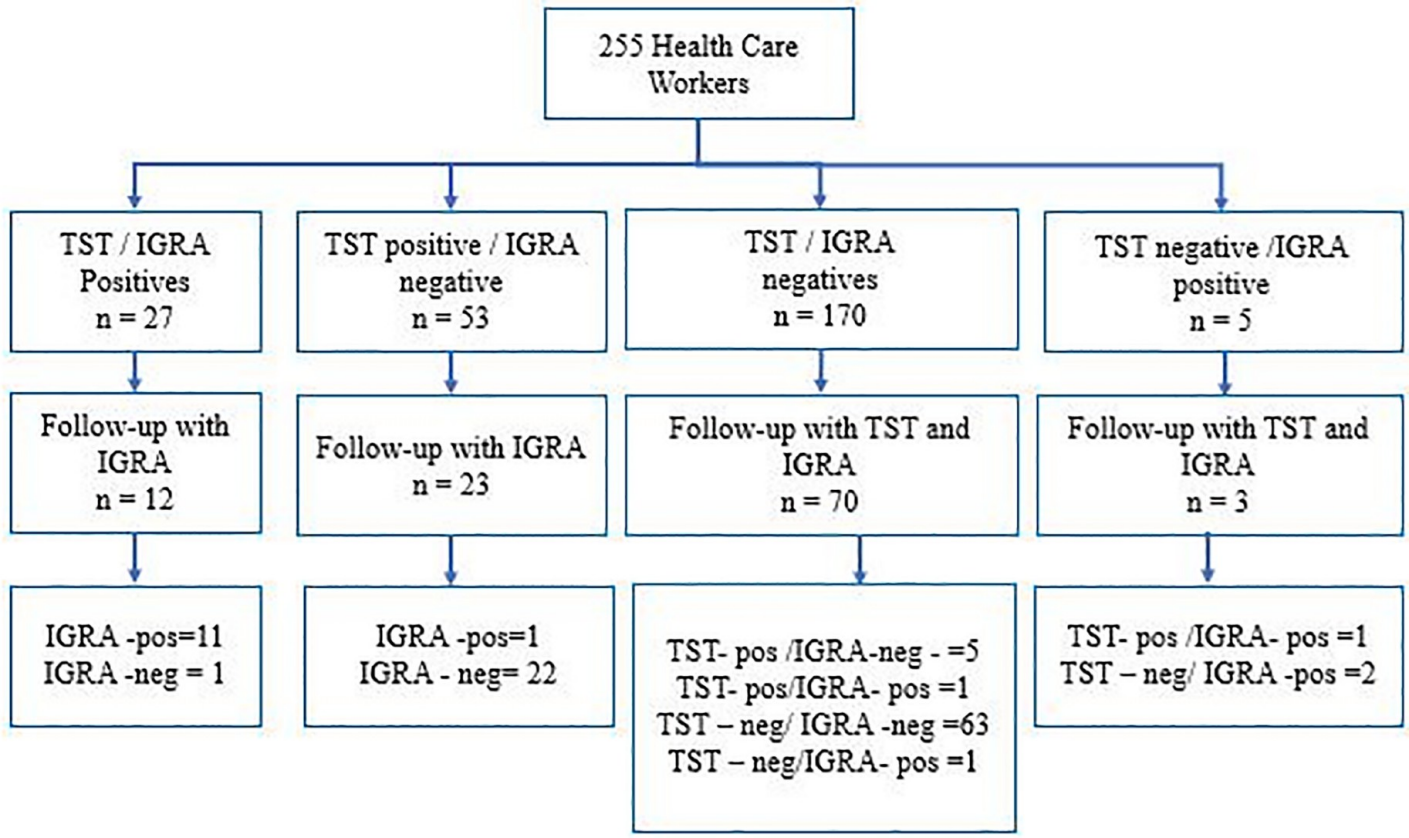
Design and description of the results of the serial tests of the study.

Furthermore, we also included the workers with a positive TST in the first phase in this second phase, regardless of the result of their IGRA tests. This group was only quantified using IFN-γ in the follow-up. Workers with baseline negative TSTs but positive IGRA tests were also included and both TSTs were administered and IFN-γ quantification performed in the follow-up (Fig 1).

### Data collection

All HCW were invited by personal letter to undertake a periodic health examination and in cases where there was evidence that there was an occupational risk of exposure to *M*. *tuberculosis*, the performance of a TST was indicated. In the Occupational Department the epidemiological-labor survey was completed, the TST test was performed and a blood sample was collected. The study was approved by the Ethics Committee of the Hospital Universitari Germans Trias i Pujol (n° PI-16-028). All HCW enrolled in this study gave a written informed consent.

#### Tuberculin skin test

The TST was administered using the Mantoux technique and interpreted according to the Spanish Society of Pulmonology Guidelines [5]. For the diagnosis of LTBI the induration diameter was measured at 48–72 hours and was considered as positive when the induration was equal to or greater than 5 mm in those not vaccinated with BCG and equal to or greater than 15 mm in those vaccinated with BCG (in all cases the presence of active tuberculosis disease was ruled out by performing a chest x-ray and if necessary a sputum microbiology).

#### IFN-γ- release assays and interpretation of results

Blood samples were collected from all selected patients using standard venipuncture methods. 1 ml of blood was collected in each of the 3 tubes. (Nil control, specific antigens ESAT-6, CFP-10 and TB.7, and mitogen tube). The tubes were incubated at 37 °C for 18 hours and the concentration of IFN- γ in the supernatants was determined by standardized techniques (QFN-G-IT, Qiagen, Düsseldorf, Germany) using specific antibodies. For this study a result was considered positive when the IU/ml of IFN-γ secreted after antigen-specific stimulation was ≥ 0,35. All the results from the nil control were subtracted from the mitogen and the antigen-specific values.

### Study variables

The main outcome variables were:

- TST measured in millimeters of induration of the transverse diameter at 48-72h of administration.
- IFN-gamma concentration expressed by T lymphocytes after stimulation with different mycobacterial antigens and amount of sensitized T cells. Measured as a quantitative variable in IU / ml and subsequently converted into a dichotomous variable (positive / negative) from a cut of 0.35 IU / ml.

In addition, other independent variables such as age, sex, current smoking status, job category, hospital service, degree of occupational exposure (high and medium/low according to the number of patients admitted to the work unit and the probability of exposure), years of professional activity, history of previous negative TST (dates), current TST (date and result), history of vaccination with BCG and dates and recent occupational exposure, were collected.

Conversion was defined as the change in TST of <5 mm to ≥5 mm in unvaccinated and the change from <15 mm to ≥ 15 mm in those vaccinated with BCG.

In the IGRA tests, the conversion of <0.35 IU / ml to ≥ 0.35 IU / ml and reversion of ≥0.35 IU / ml to <0.35 IU / ml was defined.

### Statistical analysis

The description of the quantitative variables was made using the mean and standard deviation and categorical variables using the absolute and relative frequencies. The concordance between the TST and the IGRAs tests was calculated using the Cohen's kappa coefficient. Values <0.40 were considered as poor and higher than 0.70 as good concordance. The association between both test results (IGRA and TST) and independent variables was analyzed using the chi-square test and Student's T-test. All statistical analysis was performed using the SPSS statistical package for Windows (version 25). A Multivariate Logistic Regression Analysis, with Stepwise Forward Variable Selection Method was used to analyse the variables associated with cases with discordance between the two tests. The odds ratios (OR) of discordance and the 95% confidence interval were calculated.

## Results

### Patient characteristics

The patients were predominantly female, 77.3% women and 22.7% men. The mean and standard deviation (SD) age and years of professional activity were 34.2 (SD 9.04) years and 9.5 (SD 8.5) years respectively. By professional category, 34.1% were nurses and 33.7% worked as medical doctors. Concerning the risk of tuberculosis infection at work, 26.7% of the subjects worked in areas with a medium risk, and 40.4% in other areas with a high risk (mainly Emergency department, HIV Unit, and Pulmonology Department). Positive BCG vaccination status was found in 18.4% of subjects. Previous negative TST was documented in 67.8% of participants. Table 1 summarizes the base-line characteristic of the analyzed subjects.

**Table 1:** Descriptive characteristics of the study sample (n = 255).

	n	%
**Gender (woman)**	197	77,3
**Mean age (SD) in years**	34,2 (9,0)	
**Job category**		
** Doctors**	86	33,7
** Nurse**	87	34,1
** Nursing assistants**	42	16,5
** Wardens**	9	3,5
** Technicians**	16	6,3
**Degree of exposure** ^1^		
** Low**	84	32,9
** Medium**	68	26,7
** High**	103	40,4
**BCG vaccinated**	47	18,4
**Prior negative TST**	173	67,8
**Average years of work activity (SD)**	9,5 (8,5)	

^1^ Degree of occupational exposure according to the number of patients admitted to the work unit and the probability of exposure)

### Infection prevalence

**Fig 2:**
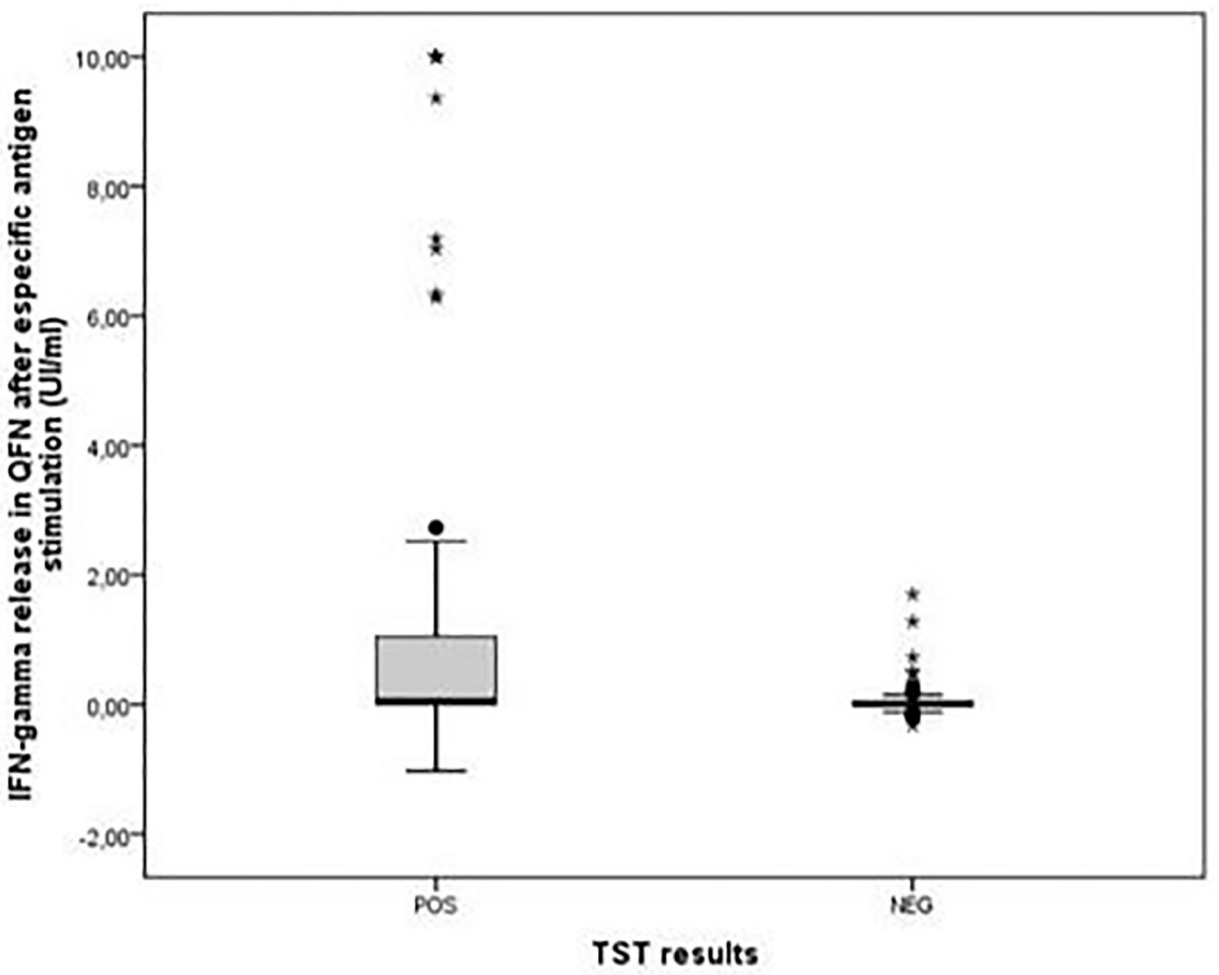
Quantification of the IGRA according to TST result.

Eighty (31.4%) and 32 (12.5%) workers were considered to be infected according to the TST and IGRA test, respectively. Fig 2 shows the distribution of IGRA test results in relation to the TST results. A higher mean of IFN-gamma concentration is observed in workers with a positive TST.

### TST and IGRA test concordance at baseline

According to the Kappa statistic, the concordance between the two tests was 0.368, with a standard error of 0.06. The highest number of discordant results showed a positive TST and a negative IGRA (53 cases); whereas only 5 cases presented a negative TST and a positive IGRA. Each case was analysed individually and both the risk of infection and the risk of disease progression were taken into account when the therapeutic option.

The kappa index was higher in workers who had not been previously vaccinated with BCG (K = 0.397, standard error = 0.074), than in those who had been previously vaccinated (K = 0.177, standard error = 0.122).

### Positive TST associated factors

The association between a higher exposure at work (p = 0.006) and the BCG vaccine positive status (p <0.001) was found to be significant (Table 2). The prevalence of infection was higher in workers with a medium or a high exposure, and also in those previously vaccinated with BCG. There was also a higher prevalence in males, although did not reach statistical significance (p = 0.062). The TST induration size was significantly higher in those previously vaccinated with BCG (mean 18.7 and SD of 4.3 mm), than in those not vaccinated (mean of 13.3 and SD of 5.5 mm) (p <0.001).

**Table 2:** Univariate analysis of the relationship of the variables with the positivity of the tests (TST and IGRA).

	Positive TST (n = 80)			Positive IGRA (n = 32)		
	n	%	p	n	%	p
**Gender**			.06			.89
** Man**	24	41,4		8	14,0	
** Woman**	56	28,4		24	12,2	
**Degree of exposure**			.006			0,056
** Low**	16	20,0		5	6,0	
** Medium**	22	27,5		9	13,4	
** High**	42	52,5		18	17,6	
**BCG vaccination**			<0,001			.001
**Yes**	28	59,6		13	27,7	
** No**	52	25,2		19	9,2	
	TST positive	TST negative		IGRA positive	IGRA negative	
**Average age in years**			.11			.337
** Mean**	35,7	33,5		35,7	33,9	
** DE**	10,4	8,3		9,8	8,8	
**Average years of work activity**			.350			.74
** Mean**	10,4	9,2		9,9	9,4	
** DE**	9,3	8,1		8,3	8,5	

### Positive IGRA test associated factors

The only factor significantly associated with a positive IGRA test result was a positive BCG vaccination status (p = 0.001). Workers with a higher level of exposure tended to present a positive result (p = 0.056) (Table 2). When stratifying by level of exposure, this association between IGRA test positivity and vaccination status remained significant only in the high exposure group (p = 0.022), but not in the medium and low exposure groups (p = 0.105 and p = 0.478 respectively).

### TST and IGRA test discordance at baseline

Discordant outcomes between tests were associated with the degree of exposure (p = 0.003) and BCG vaccination status (p <0.001). In the HCW from the group with a higher risk of exposure 33.3% of the results were discordant and, in the BCG vaccinated group we found 44.7% of cases had discordant results. In the multivariate logistic regression, using the pairs of concordant results as a reference (the two positive tests or the two negative ones), high degree of exposure (OR = 3,04; 95% Confidence Interval of 1.4–6.5) and BCG vaccination (OR = 3.5; 95% CI of 1.7–7.0) were maintained as predictive factors of discordance.

Induration TST size was higher in concordant cases (mean and SD: 18.3, 5.3 mm) than in the discordant cases (mean and SD: 13.6, 5.2 mm) (p <0.001). This analysis stratified by BCG vaccination also showed major induration TST size in concordant cases.

### Results of the follow-up study

Out of the 108 workers followed up from the same cohort, thirty-five presented positive baseline TST and 15 had positive baseline IGRA. In 15 workers a third follow-up was carried out.

During the follow-up period, seven workers presented TST conversion. One of these conversions was also confirmed with an IGRA test. Treatment of LTBI was offered to all positive cases, but only two of them started taking the treatment. All baseline IGRA results were below 0.20 IU/ml, except in one case, which presented a negative baseline TST and a 0.51 IU/ml IGRA result afterwards. Except for one, all TST indurations changed from 0 (baseline) to >10 mm after conversion. In 2 cases of conversion with TST alone, a third follow-up with IGRA was performed, which was also negative (Table 3).

**Table 3:** Description of conversions and reversions.

	Gender	Age	BCG	Work area	TST Baseline (mm)	IGRA Baseline	2ª TST (mm)	2° IGRA	3° IGRA
**Conversions**									
Case 1	Woman	34	Yes	High	0	NEG	20	NEG (0,03)	NEG (0,00)
Case 2	Woman	51	No	High	0	NEG	8	NEG (0,01)	NEG (0,00)
Case 3	Man	38	No	Low	0	NEG (0,04)	15	POS (0,5)	
Case 4	Woman	25	No	Low	0	NEG (0,03)	10	NEG (0,03)	
Case 5	Woman	25	No	High	0	NEG (0,02)	18	NEG (0,01)	
Case 6	Woman	44	Yes	High	0	POS (0,51)	20	POS (0,66)	
Case 7	Man	49	No	High	0	NEG (0,04)	20	NEG (0,07)	
Case 8	Man	25	No	High	7	NEG (0,22)	-	POS (0,82)	
Case 9	Woman	38	No	Medium	0	NEG (0,06)	0	POS (0,38)	
**Reversions**									
Case 10	Woman	27	No	High	NEG	POS (1,70)	0	NEG (0,03)	
Case 11	Woman	51	No	Medium	POS (18)	POS (0,80)	-	NEG (0,23)	

NEG: negative; POS: positive

There were 2 cases of conversions for subjects who only showed positive with the IGRA: one case with positive baseline TST of 7 mm and another with negative baseline TST but with follow-up IGRA of 0.38 IU / ml.

There have been only 2 reversions with the IGRA, one with an 18 mm basal TST. In these 2 cases of reversions, the baseline IGRA was above 0.70 IU/ml (1.70 and 0.80). In both cases the HCW refused the LTBI treatment.

No case so far has developed tuberculosis.

## Discussion

To our knowledge only a few studies of serial testing in HCW have assessed subjects by both TST and IGRA tests simultaneously. Most of the studies presented a cross-sectional design where both tests were performed at the same time, analyzing factors associated with concordance and discordance [15, 16]. Our study adds more information about conversions and reversions in this risk group and provides more evidence of the performance of the IGRA test in workers vaccinated with BCG.

Taking into account positive TST or IGRA results, the prevalence of infection found in this study was 31.4% and 12.5%, respectively. This prevalence is similar to that found in other studies carried out in Catalonia [17, 18] and also in other studies that describe this data in countries of low and intermediate income [2, 19, 20]. Factors associated with the positivity of either TST or IGRA test were BCG vaccination status and degree of occupational exposure to tuberculosis. These factors have also been found in other studies [2], where occupational factors were independent factors associated with tuberculosis infection, especially those that involved more direct contact with a TB patients or prolonged exposure.

A curious fact is that in the initial analysis BCG vaccination was found to be associated with the positivity of the IGRA (27.7% of those vaccinated were positive versus 9.2% of those not vaccinated). But stratifying by degree of exposure, this association only remained for the high degree of exposure. 40% of workers in the sample studied had a high degree of exposure.

Factors associated with test discordance were BCG vaccine status and the degree of occupational exposure. Similar results were published by Adams et al., who found BCG vaccination status and years of exposure as predictors of TST and IGRA test discordant outcomes.

Those workers that had previously been vaccinated with BCG presented a lower concordance between tests. The majority of discordant cases have given a positive TST and negative IGRA (58 cases), while negative TST and positive IGRA has only been found in 5 cases. In vaccinated workers 52.9% of negative IGRA had a positive TST, while in unvaccinated only 18.7% of negative IGRA had a positive TST. In our study we have detected a relationship between the degree of exposure and the baseline discrepancies

According to the TST results, the incidence of tuberculosis infection during the study period was 9.9% (7/73), higher than previously reported in other studies. In relation to the IGRA results, the incidence of infection was 3.2% (3/93), in line with data reported in studies from similar countries. The recent review by Apriani et al [2]. presented more information from countries of low or intermediate economic income. The earlier review by Uden et al [19]. found a higher incidence in these countries.

TST conversions that have not been confirmed with IGRA tests may be explained by TST that are false positive in the follow-up (booster, vaccinated with BCG). Only one TST conversion was associated with an IGRA conversion. It should be borne in mind that in two cases of conversions with the TST alone, a third follow-up was carried out and the IGRA remained negative. There was 1 case of negative initial TST and positive baseline IGRA and that made a TST conversion at follow-up.

In the case of the 2 conversions with the IGRA only, it should be taken into account that in one case the follow-up IGRA was 0.38 IU/ml, a value very close to the cut-off 0,35 IU/ml. The other case had a baseline IGRA of 0.22 IU/ml, this is in the grey zone (between 0,20 and 070 IU/ml), indicating that could be a positive baseline.

Our series presented only two cases of reversion, which contrasts with the higher percentage of reversions that was described by Schablon et al [21] in Germany. Previous studies have suggested the existence of a gray area in the results of the IGRA [22, 23]. This area, which ranges from 0.20 to 0.70 IU / ml, should be taken into account when interpreting the results from serial tests, especially in low-incidence countries. In the study by Pai et al [22], conducted in HCW, the reversion of the IGRA results resulted in limit values of the positive results of the IGRA, although they also did not rule out the possibility that the reversals could reflect a spontaneous elimination of tuberculosis infection. The two reversals found in our study had baseline values out of the grey zone (0.80 and 1.7 IU / ml). One of them had a baseline negative TST, with a subsequent result of 0 mm. The other case presented a baseline TST of 18 mm and the follow-up with IGRA result was found in the gray zone (0.23 IU / ml).

Currently, there is a new version of the IGRA test, in which uses two different stimulated types of ESAT-6 and CFP-10 to stimulate the CD4 and CD8. Recent studies do not show significant differences between the previous and this new version of the test in terms of the number of positive outcomes when studying *M*. *tuberculosis* infection [24].

This study has some limitations: Firstly, due to organizational factors there were some variations in the periodicity of health exams by the Occupational Department during the follow-up period. This limitation could affect in the diagnosis of some cases of possible recent infection. Secondly, the low number of conversions and reversions did not allow for a generalization of the results. For this same reason it was not possible to study the factors associated with the gray area of 0.2–0.7.

Finally, it should be noted that the last update of the CDC guidelines in 2019 [25] suggests that in situations where the risk of infection is low, it is not recommended to routinely perform serial screening of HCW. Only at hiring, and in high-risk workers. In the rest of cases only if there has been exposure at work. However, it is highly recommended to strengthen health education in order to reduce the risk of occupational exposure, as well as treating all LTBI in workers with untreated tuberculosis infection, unless the medication is contraindicated. WHO also recommends not using serial screening with IGRA in countries with low or intermediate incomes, due to the high number of conversions and reversions of these tests [26]. In our study only 1 of the 7 conversions with TST were confirmed with the IGRA test, and 4 discordances in the follow-up were in high-degree risk HCW. Given that it is currently recommended to only perform screening for HCW with a high level of risk, even though we only have low number of cases, the results suggest that it is important to continue with the 2-step strategy in these cases.

## Conclusion

In summary, in this study, not all TST conversions were confirmed when using the IGRA test, which highlights the importance of the 2-step strategy (TST followed by confirmation with IGRA). Either diagnostic screening test must be interpreted according to other factors, both labor and non-labor, in order to decide the subsequent therapeutic action. Our conclusions should be confirmed in studies with a higher number of tested subjects and also longer follow-up time.

## Supporting information

S1 File (XLSX)
